# Synthesis of glycerol carbonate from glycerin with CaCO_3_ in a batch reactor

**DOI:** 10.1080/15685551.2022.2037215

**Published:** 2022-02-10

**Authors:** Ana Beatriz Morales Cepeda, Luis A. Macclesh del Pino, Claudia Esmeralda Ramos Galvan, Eric Joaquín González Pedraza, Luciano Aguilera Vazquez

**Affiliations:** Tecnológico Nacional de México/ Instituto Tecnológico de Ciudad Madero, Parque Tecnia (Pequeña y Mediana Industria), Bahía Aldhair, México

**Keywords:** Glycerol Carbonante, batch reactor, synthesis

## Abstract

In the present work, a reaction methodology was implemented using a batch reactor, which synthesized glycerol carbonate (GC) using glycerin and CaCO_3_. A crystallographic analysis of CaCO_3_ was performed to determine its crystalline form. The obtained product was characterized by infrared spectroscopy, thermogravimetric analysis and nuclear magnetic resonance (^1^H and ^13^C). Our analysis demonstrated that the obtained product with the implemented reaction methodology has GC, FTIR showed the signals of the carbonyl groups, and the NMR spectrum confirmed the presence of cyclic carbonate structure in addition to linear carbonates. The thermogravimetric study showed that the thermal stability of the product is highly similar to that reported for GC. These results exhibit that the synthesis process produces linear and cyclic carbonates.

## Introduction

**Scheme 1.**
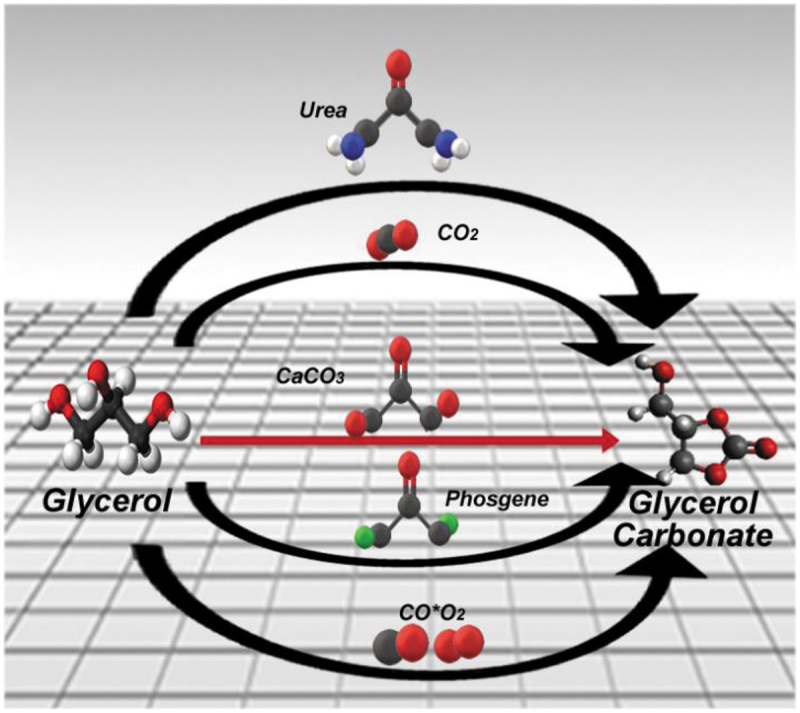
Synthesis routes of glycerol carbonate.

The development of environmentally friendly processes which allow the use of materials considered as waste, is a common area of interest for various research groups. This has promoted the development of different synthetic routes [1]; particularly, a route to obtain monomers with low environmental impact. In recent years, a boost in the biodiesel-producing industries has resulted in an overproduction of glycerin (a by-product of this process), resulting in a remarkable price drop. Production in United States of America exceeds 350,000 tons per year [2]. One of the chemical derivatives produced from glycerin is glycerol carbonate (GC), a high-value product with a wide range of applications [3,4]. Glycerol carbonate has an excellent chemical stability, it does not present flammability and is soluble in water; the latter goes hand in hand with its zero impact on health and the environment due to its low toxicity (not mentioned R-Phrase in its Material Safety Data Sheet). Due to its water solubility, it possesses good biodegradability and low volatility (bp 110–115°C a 0.1 mmHg) [5]. There are different pathways for the synthesis of glycerol carbonate [6] (Scheme 1).

Glycerol carbonate has several applications: as a chemical intermediate in the production of polymers [7], as a curing agent [8], a surfactant, as the electrolytes of choice in the production of lithium-ion batteries and as a carrier solvent [9]. Glycerol carbonate can be obtained by transesterification with a cyclic carbonate such as ethylene carbonate or other alkylene carbonates [10]; Marya *et al*. [11] reported the synthesis of glycerol carbonate and diethyl carbonate catalyzed by hydrotalcite compounds. Recently, Angela Dibenedetto *et al*., experimented with direct carboxylation using CO_2_, developing a synthetic pathway using CO_2_ and a metallic catalyst [12]. Within the synthetic processes to obtain glycerol carbonate, the synthesis through CO_2_ has been carried out in both homogeneous and heterogeneous catalysis. The first experiments were performed in the presence of metal alkoxides such as n-Bu_2_Sn(OMe)_2_ and n-Bu_2_SnO [13–14]. In addition, López Ortiz *et al*. [15], performed a thermodynamic analysis and process simulation for H_2_ production by dry reforming of ethanol using CaCO_3_ as the CO_2_ source for the reaction system, an innovative approach in the use of a solid carbonate to serve as a source of CO_2_ to produce hydrogen-synthesis gas (H_2_-syngas). In order to avoid toxic agents, the use of ethanol was proposed for the synthesis of GC coupled with calcium carbonate, where a closed system (batch reactor) was implemented, in which the CO_2_ released by CaCO_3_ was reused during the reaction.

## Methodology

**Scheme 2.**
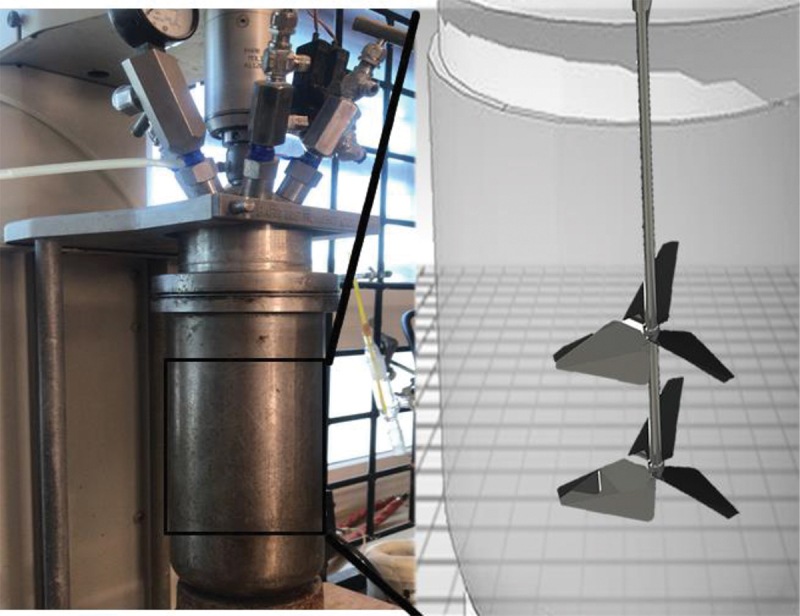
Batch reactor with double propeller stirrer.

In order to synthesize the glycerol carbonate, Omyacarb® UF calcium carbonate (Omya, Switzerland) was employed, with a particle size of 0.7 μm, without any surface modification treatment. Additionally, we used > 99% purity glycerin (Sigma Aldrich, United States) and 96% purity ethanol (Fermont, Mexico). This reaction was performed in a 2 L Parr reactor as shown in Scheme 2. As it is known, CaCO_3_ decomposes at very high temperature, forming CO_2_ which is needed for the synthesis. 500 ml of glycerin was employed to react with CaCO_3_ in a 20 % ratio (glycerin/ CaCO_3_). In order to facilitate the process of decomposition of CaCO_3_, ethanol was incorporated at a 1:0.5 ratio, concerning the volume of glycerin. The glycerin/ethanol/CaCO_3_ mixture has been studied previously by Dang *et al*. [16] to produce synthetic gas, with the reaction between ethanol and CaCO_3_ being:

$$C_{2}H_{5}OH+CaCO_{3}\to CO_{2}+2CO+CaO+3H_{2}$$

As shown in Scheme 2, the reactor has a double propeller stirrer, which adds CaCO_3_ and ethanol into the glycerin. Since the low solubility of CaCO_3_ requires vigorous agitation. The synthesis was performed in the time span of 8 hours, under similar conditions at a temperature of 250°C and a pressure of 45 PSIA.

After the reaction, the obtained product was filtered and centrifuged in order to remove all solids. After this, all volatile compounds were removed using a rotary evaporator. Finally, ethyl acetate was employed to extract the organic fraction in the solution using a separation funnel, and later the ethyl acetate was recovered by rotary evaporation.

Next, the identification of functional groups was performed using a FTIR spectrophotometer with diamond ATR (Spectrum 100, Perkin Elmer, USA) to determine the presence of the GC, through the identification of the expected functional groups. The thermogravimetric analysis was carried out on a TGA/DSC (Model SDT Q600, TA Instruments, USA), and it was possible to observe the degradation temperature of the product obtained from the reaction. Calcium carbonate was analyzed by X-ray diffraction to determine its crystalline structure and purity. Finally, through a proton NMR analysis (Advance III HD 400 MHz equipment Bruker, USA), the structure belonging to the GC was identified in the obtained product.

## Results

Glycerin is chemically similar in its hydrocarbon chain with three functional OH groups. Therefore, if the calcium carbonate performs the Ca^+2^ ion displacement at constant pressure and temperature [17,18], we had the opportunity to achieve the reaction.

**Figure 1.**
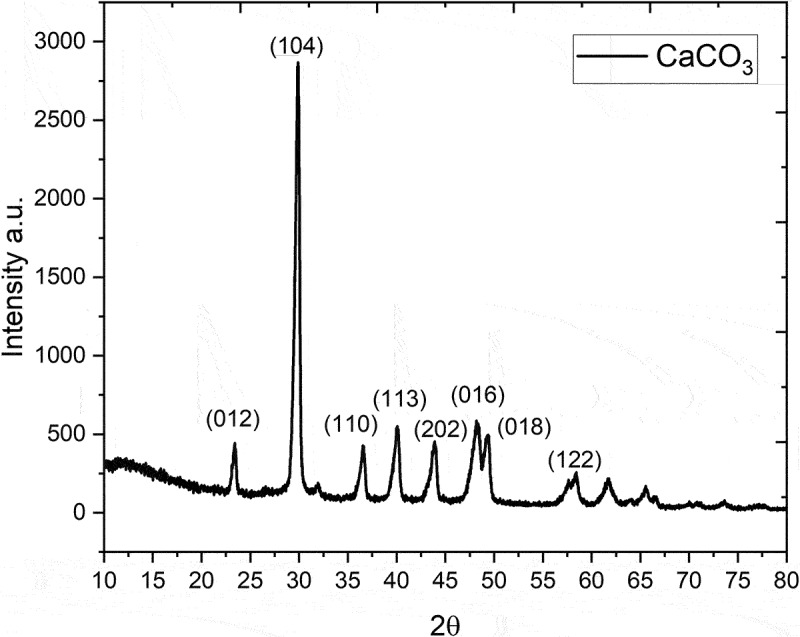
Diffraction patterns of CaCO_3._

Calcium carbonate (CaCO_3_) is the most abundant mineral in nature, making it one of the most economical inorganic materials [19]. X-ray diffraction was used to determine the structure and purity of CaCO_3_. Our study shows that there are particles with high crystallinity. In Figure 1 Diffraction patterns are observed in 2Ѳ in 23°, 29.8°, 36.7°, 39.9°, 43.5°, 48.25°, 49.6° and 58.3° which represent lattices (0 1 2), (1 0 4), (1 1 0), (1 1 3), (2 0 2), (0 1 6), (0 1 8) and (1 2 2), respectively. The signs are present at 29.8° and 43.5° CaCO_3_ in the form of calcite [20]. Sharp signals are presented, which can be indexed in the CaCO_3_ diffraction chart (JCPDS88-1808). No signs of impurities were found.

### NMR analysis

**Figure 2.**
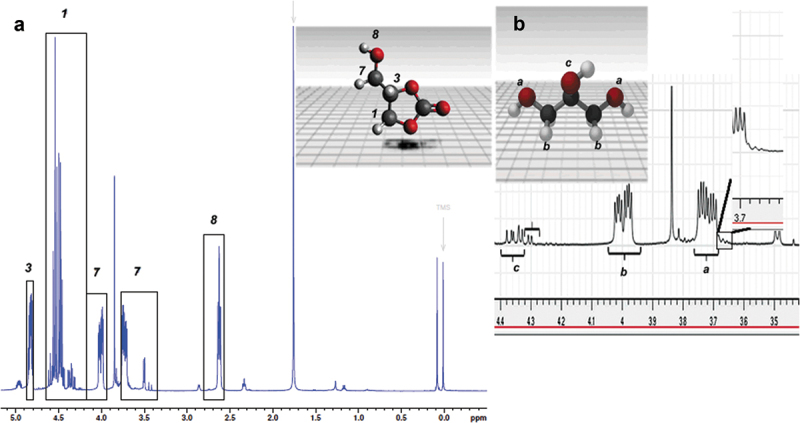
NMR ^1^H spectrum of product.

**Figure 3.**
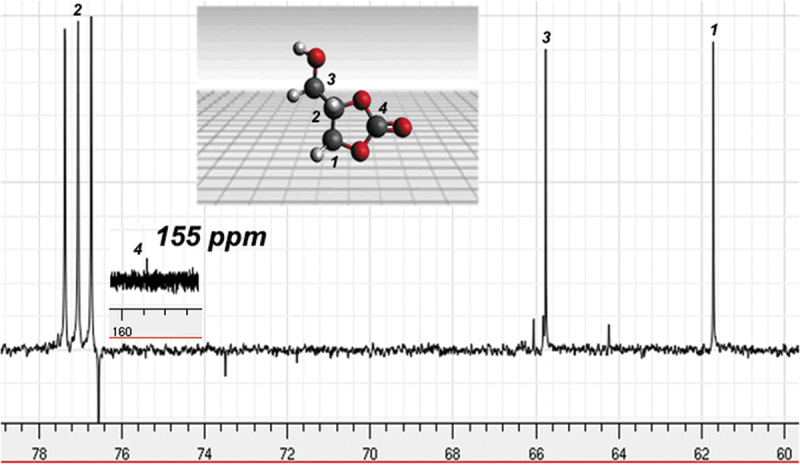
NMR ^13^C spectrum of the product.

**Scheme 3.**
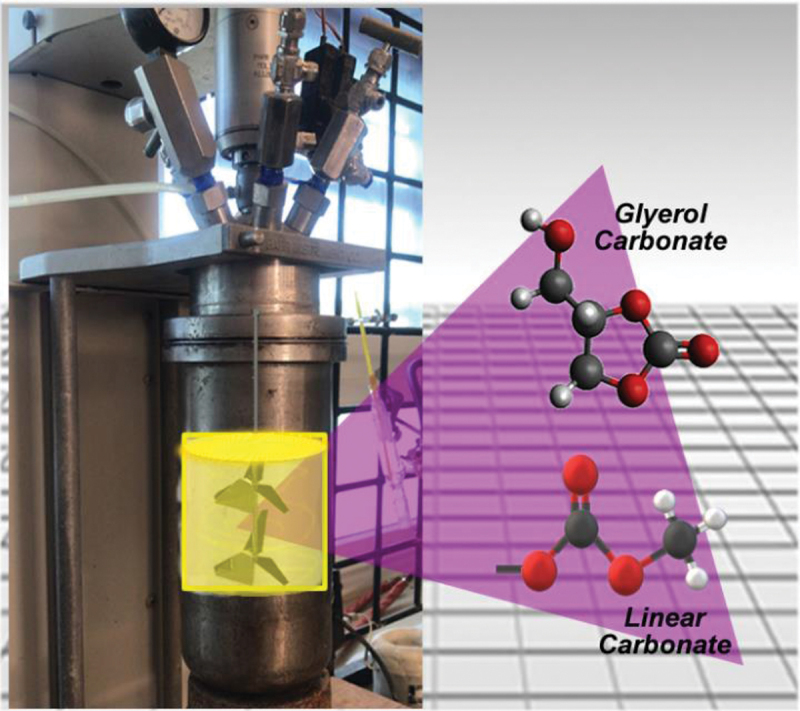
Cyclic and linear structures formed.

Product formation was confirmed through proton NMR. ^1^H NMR has been used to determine the chemical structure of the GC molecule, previously [21,22]. In the ^1^H NMR spectrum (Figure 2(a)) we can see the mixture of GC and glycerin, we can notice in Figure 2(b) the signals in the 4.4–3.6 ppm regions of the proton c, b, a, belonging to glycerin, there is also an overlap of the a1 signal of the glycerol carbonate. Signals belonging to linear chains are observed at 4.3 ppm, 3.69 ppm, and 3.68 ppm; these signals have been previously reported by Acemoglu *et al*. [23]. The glycerol carbonate NMR ^1^H are ^1^H (DMSO, 500 MHz), δ (ppm), 4.41 (H_1_, CH_2_), 4.83 (H_1_, CH) 3.63, 4.01 (H_1_, CH-CH_2_), 2.63 (H_1_, OH). In Figure 3, the ^13^C spectrum of the product is observed, and it presents signals from cyclic carbon atoms, as well as linear units. Only a few representative standards of oligoglycerols are described in the literature [24–27]. The spectrum presents the signals 2 = 77 ppm, 3 = 65.6 ppm, 1 = 65.2 ppm, 4 = 155 ppm characteristic of glycerol carbonate; these signals have been reported by Aresta *et al*. [28]. Linear carbonate signals are located at 64 ppm, 76 ppm for linear carbon atoms of 1–3 units. This indicates that the synthesis pathway facilitates the formation of cyclic carbonates such as glycerol carbonate and linear carbonates, as shown in Scheme 3.

**Figure 4.**
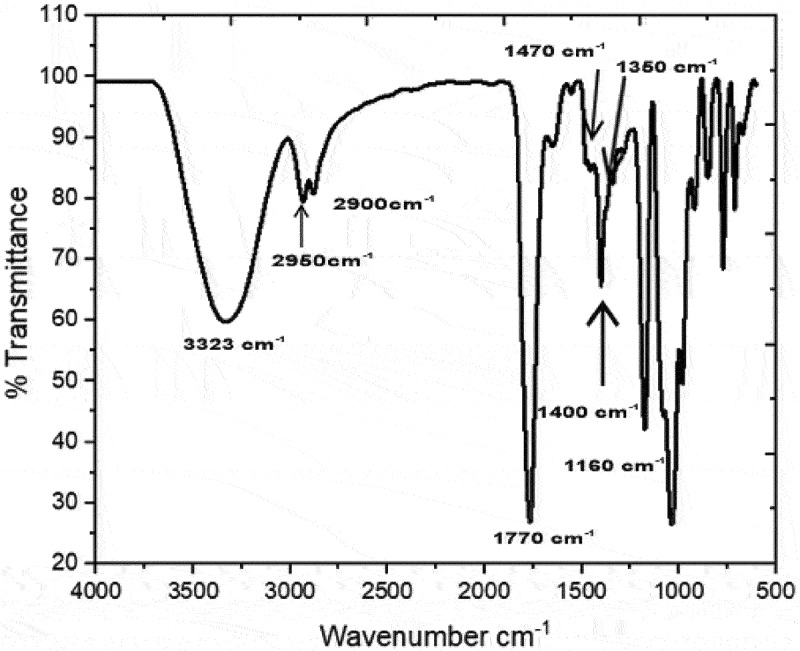
FTIR spectrum of the product obtained after reaction.

After the synthesis process, the sample was analyzed by FTIR, in Figure 4, the product spectrum obtained as a result of the reaction is observed, in which the bands 3323 cm^−1^ and 1400 cm^−1^ are noticed, due to the O-H vibrations of the 2-hydroxymethyl chain [29]. In addition, the absorption bands close to 2950 and 2900 cm^−1^ are present due to the vibrations of the CH_2_ and CH [30] bonds of the O-methylene and O-methylidyne cyclic groups belonging to the carbonate. At 1770 cm^−1^, the absorption band originated by the stretching of C=O groups is appreciated [31]; this double stretching is due to the formation of linear carbonate groups and cyclic carbonates [32]. Around 1170 cm^−1^ and 1030 cm^−1^, the signal of the C–C and C–O stretching, respectively, of the chain, two hydroxyethyls appear [33]; all these signals confirm the presence of glycerol carbonate in the product obtained from the reaction. It should be mentioned that the presence of glycerol in the product is notorious due to the signs at 1480 cm^−1^, 1340 cm^−1^, and 1160 cm^−1^ [34]. Indran *et al*. [35] reported similar signals after their synthesis route.

### Thermogravimetric analysis

**Figure 5.**
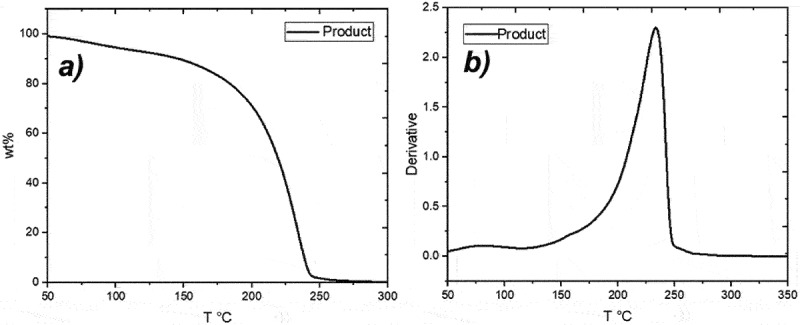
Product thermogravimetric curves.

The thermogravimetric study showed the thermal stability of the product; in Figure 5(a) the thermogram is appreciated. The CG has excellent chemical stability, Flash Point > 204° [5]; the latter is observed in the curve of the TGA (black line), which exhibits a 10% weight loss between 50°C and 100°C, probably due to remnants of both water and glycerin, next to a noticeable weight loss at 170°C and the major weight loss occurred between 200°C and 245°C, a clear indication of the breakdown of glycerol carbonate. In Figure 5(b), the derivative of weight percentage shows again that the total loss occurs below 250°C, something plausible due to the previously mentioned weight loss due to moisture and remaining glycerin.

## Conclusion

Our results show that the reaction process synthesized glycerol carbonate in a batch system implementing CaCO_3_ and a reaction time. The FTIR study showed the presence of carbonyl groups in the synthesized product, while the thermogravimetric study showed that the product has thermal stability close to that of GC, with magnetic resonance confirming the structural presence of GC, in addition to the presence of glycerin. Similarly, the NMR spectra revealed the presence of the cyclic structure of glycerol carbonate, in addition to the presence of linear carbonate group chains. The synthesis process proved to be suitable for producing a monomer of interest, which by known polymerization routes can generate a product of high economic value.
